# Resource flows for health care: Namibia reproductive health sub-accounts

**DOI:** 10.1186/1755-7682-4-41

**Published:** 2011-12-24

**Authors:** Thomas Mbeeli, Muine Samahiya, Nirmala Ravishankar, Eyob Zere, Joses M Kirigia

**Affiliations:** 1 Ministry of Health and Social Services, Windhoek, Namibia; 2 Health Systems 20/20, Abt Associates, Bethesda, USA; 3 World Health Organization, Regional Office for Africa, Brazzaville, Congo; 4 Centre for Health Policy and Capacity Development, FHI Development 360, Washington DC, USA

## Abstract

**Background:**

Implementing initiatives to achieve the targets of MDG 5 requires sufficient financial resources that are mobilized and utilized in an equitable, efficient and sustainable manner. Informed decision making to this end requires the availability of reliable health financing information. This is accomplished by means of Reproductive Health (RH) sub-account, which captures and organizes expenditure on RH services in two-dimensional tables from financing sources to end users. The specific objectives of this study are: (i) to quantify total expenditure on reproductive health services; and (ii) to examine the flow of RH funds from sources to end users.

**Methods:**

The RH sub-account was part of the general National Health Accounts exercise covering the Financial Years 2007/08 and 2008/09. Primary data were collected from employers, medical aid schemes, donors and government ministries using questionnaire. Secondary data were obtained from various documents of the Namibian Government and the health financing database of the World Health Organization. Data were analyzed using a data screen designed in Microsoft Excel.

**Results:**

RH expenditure per woman of reproductive age was US$ 148 and US$ 126 in the 2007/08 and 2008/09 financial years respectively. This is by far higher than what is observed in most African countries. RH expenditure constituted more than 10-12% of the total expenditure on health. Out-of-pocket payment for RH was minimal (less than 4% of the RH spending in both years). Government is the key source of RH spending. Moreover, the public sector is the main financing agent with programmatic control of RH funds and also the main provider of services. Most of the RH expenditure is spent on services of curative care (both in- and out-patient). The proportion allocated for preventive and public health services was not more than 5% in the two financial years.

**Conclusion:**

Namibia's expenditure on reproductive health is remarkable by the standards of Africa and other middle-income countries. However, an increasing maternal mortality ratio does not bode well with the level of reproductive health expenditure. It is therefore important to critically examine the state of efficiency in the allocation and use of reproductive health expenditures in order to improve health outcomes.

## Background

With less than four years left to achieve the Millennium Development Goals (MDGs), progress towards the targets of the health-related MDGs has not been promising in the African Region. This is more pronounced in the MDG 5 target of reducing the maternal mortality ratio by three-quarters, between 1990 and 2015. In sub-Saharan Africa, the maternal mortality ratio decreased from an average of 870 per 100,000 live births in 1990 to 640 per 100,000 births in 2008 [1], translating to average annual rate of reduction of 1.7%, which is much lower than what is required to achieve the target of maternal mortality reduction.

In Namibia, despite an increasing and relatively good coverage of maternal health interventions such as antenatal care and skilled birth attendance, the maternal mortality ratio increased from 271 per 100,000 live births in the period 1991-2000 to 449 per 100,000 live births in the period 1998-2007. While the sampling errors around each of the estimates are large, the confidence intervals around the estimates from the 2000 NDHS and 2006-07 NDHS do not overlap. Thus, it is possible to say with reasonable confidence that maternal mortality in Namibia increased in the recent past [2].

Investing in maternal health is urgent not only because giving life should not result in death, but also because women's health is critical to sustainable socio-economic development in Africa. Furthermore, investing in maternal health is a way to improve health systems overall, which benefits the entire population of a country [3]. In cognizance of this, the Millennium Declaration in 2000 by the Heads of State of the United Nations Member States put improving maternal health as one of the Millennium Development Goals-MDG 5. This MDG has two targets: (1) reducing by three quarters, between 1990 and 2015, the maternal mortality ratio; and (ii) achieving by 2015, universal access to reproductive health services.

To reverse the increasing trend in maternal mortality and achieve the MDG 5 target the Government has embarked on a number of initiatives, including the development and implementation of a Roadmap for Accelerating the Reduction of Maternal and Neonatal morbidity and mortality and launching of the Campaign on Accelerated Reduction of Maternal Mortality in Africa (CARMMA) in 2009 [4,5]. Implementation of the Roadmap, which covers the period 2009-2014, is estimated to cost about US$ 717.2 million [6]. It has to be noted that this is the cost of implementing activities to accelerate progress towards achieving the targets of both MDGs 4 and 5. Given historical expenditure patterns, mobilizing resources to cover the cost of implementing the CARMMA may be an enormous challenge.

Implementing the above initiatives to achieve the targets of MDG 5 requires sufficient financial resources that are mobilized and utilized in an equitable, efficient and sustainable manner [7]. Informed decision making requires the availability of health financing information which can address issues such as:

how much money is currently being spent on reproductive health (RH) services;

who finances RH services;

Who manages RH finances;

What RH services and inputs are purchased with the amount of money spent; and

who benefits from RH expenditure

A tool that is commonly used to address the above issues is the RH sub-account within the National health Accounts (NHA) framework. The RH sub-account captures and organizes expenditure on RH services in two-dimensional tables from financing sources to end users, and is comprehensive in scope as it includes public, private and donor funds flows [7].

The aim of this report is to provide an overall picture of reproductive health financing in Namibia. The specific objectives are to:

quantify the total expenditure on reproductive health services; and

examine the flow of RH funds from sources to end users

### Brief country profile

Namibia is situated in southwestern part of Africa and covers a land area of approximately 824,000 square kilometers. The country's population is estimated at about 2.1 million in 2009 [8].

Namibia is an upper middle income country [9] and one of those with the highest income inequality in the world [10]. Table 1 presents data on selected health and development indicators of Namibia [2,6,10-12].

**Table 1:** Namibia: selected health and development indicators

Indicator	Value
GDP per capita, 2008 (US$)	4,149
Income Gini index, 2000-2010	74.3
Human Development Index (HDI), 2010	0.606
Life expectancy at birth, 2007 (years0	59
Infant mortality rate per 1,000 live births, 2002-2006	46
Under-five mortality rate per 1,000 live births, 2002-2006	69
Total number of women age 15-49 years (2007-2008)	438,650
Total number of women age 15-49 years (2008-2009)	450,055
Annual number of births, 2009	59,000
Maternal mortality ratio per 100,000 live births (1998-2007)	449
Adult (15-49) years HIV prevalence rate, 2008 (%)	15.3
Antenatal care from skilled provider-4+ visits, 2006-2007 (%)	70.4
Percentage of deliveries by a skilled provider, 2006-2007 (%)	81.4
Health expenditure per capita 2008/09 (US$)	268
Physician per 10,000 population, 2000-2007	3
Nursing and midwifery personnel per 10,000 population, 2000-2007	31
Hospital beds per 10,000 population, 2000-2008	94.6

As can be seen from Table 1 the country is better off than many countries in sub-Saharan Africa in terms of inputs such as per capita spending on health and human resources for health densities and in terms of utilization of services such as antenatal care and skilled attendance at birth. However, despite these relatively favorable indicators, the maternal mortality ratio has been on the increase.

## Methods

### Data sources

The RH sub-account, which was part of the general NHA study [6], covered two financial years: 2007/2008 and 2008/2009. Primary data were collected from employers, medical aid schemes, donors and government ministries. Sources of secondary data included: estimates of Revenue and Expenditures for 2007/08 and 2008/09; Medium-Term Expenditure Frameworks, 2007/08 and 2008/09; Namibia National Accounts, 2008; health Facility Census, 2009; Maternal and Child Health Road Map Costing; Namibia household income and expenditure survey (NHIES), 2003/04; Namibia Demographic and Health Survey (NDHS), 2006/07; WHO health financing database; essential indicators Report, 2007/08; and Namibia consumer price index, March 2010.

### Data collection

Questionnaires were developed and tested for the primary data collection based on the experience of previous rounds of NHA and that of other countries. Enumerators were trained for two days to administer the questionnaires in the capital city, Windhoek, where the majority of the responding organizations are based. The NHA team of the Ministry of Health and Social Services (MOHSS) administered the questionnaires to organizations in the coastal towns of Swakopmund and Walvis Bay. Questionnaires were e-mailed to respondents elsewhere.

Prior to embarking on the data collection exercise, two stakeholder sensitization workshops were conducted in order to increase awareness on the importance of tracking finances for health and dispelling misconceptions on the nature and purpose of the information requested from the respondents.

### Sampling

All non-government organizations and donors that are active in the health domain and all of the 11 medical aid schemes in Namibia were surveyed. However, for the employer survey, 100 companies were selected out of a total of 273 companies with a staff complement of 65,412. Five strata were constituted on the basis of the concentration of the companies in the country: Windhoek, Walvis Bay, Swakopmund, Luderitz and rest of the country. Companies were selected from each stratum using simple random sampling proportionate to size. The four largest cities in Namibia-Windhoek, Walvis Bay, Swakopmund and Luderitz accounted for about 86% of the companies and 84% of the total employees.

### Data analysis

The row data were screened in a validation workshop to identify gaps and inconsistencies. Trained data entry clerks captured the data using a data screen designed in Microsoft Excel, Secondary data were collected and used to determine ratios and in populating the NHA tables. The following four tables were produced showing resource flows between institutional health system actors and functions:

RH expenditure by Financing Sources and Financing agents ((FS × HF);

RH expenditure by Financing Agents and Providers (HF × HP);

RH expenditure by Providers and Functions (HP × HC); and

RH expenditure by Financing Agents to Functions (HF × HC).

As no household health expenditure and utilization survey was carried out, estimates of household out-of-pocket expenditure on health were made using data from existing surveys. Estimation was done using health expenditures reported in NHIES 2003/04 after adjustments for inflation and population growth. The ratio for RH was determined using data from the Service Provision Assessment (SPA) and the Maternal and Child Health (MCH) Road Map Costing. Utilization data from the SPA were used with cost information from the MCH costing to calculate total OOP expenditure on RH-related services.

The following RH financing indicators were computed using both the primary and secondary data collected:

• General indicators:

- Total RH expenditure (TRHE)

- RH expenditure per woman of reproductive age (15-49 years)

- RH expenditure as a percentage of GDP

- RH expenditure as a percentage of total health expenditure (THE)

• Financing source indicators:

- public contribution as a percentage of TRHE

- private company contribution as a percentage of TRHE

- household contribution as a percentage of TRHE

- donor contribution as a percentage of TRHE

• Household health expenditure indicators:

- Total RH household spending as a % of TRHE

- OOP expenditure as a percentage of total household expenditure on RH

- OOP expenditure as a percentage of TRHE

- OOP expenditure per woman of reproductive age

• Financing agent indicators:

- percentage of RH funds managed by public entities

- percentage of RH funds managed by private entities

- percentage of RH funds managed by donors and NGOs

• Provider indicators:

- expenditure by public facilities as a percentage of TRHE

- expenditure by private facilities as a percentage of TRHE

- expenditure by dispensing chemists as a percentage of TRHE

- expenditure by provision and administration of public health programs as a percentage of THE

• Functional indicators:

- expenditure on inpatient curative care as a percentage of TRHE

- expenditure on outpatient curative care as a percentage of TRHE

- expenditure on Pharmaceuticals from retail pharmacies as a percentage of TRHE

- expenditure on public health programs as a percentage of TRHE

- expenditure on health administration as a percentage of TRHE

### Definition of terms

To facilitate understanding of the report, the core terms used in NHA are defined and adapted for RH below [13].

i. **Financing sources (FS)**: Institutions or entities that provide the funds for reproductive health care. They are the originators of the funds (e.g. Ministry of finance, households, donors etc).

ii. **Financing agents (HF)**: Institutions or entities that channel funds provided by the financing sources and use those funds to pay for or purchase the activities inside the RH sub-accounts boundary. Examples of financing agents include Ministry of Health, insurance companies and households that purchase RH services.

iii. **Providers (HP)**: Entities that receive money in exchange for or in anticipation of producing the activities inside the RH sub-accounts boundary. They are the providers of RH services. Examples of providers include hospitals, clinics, pharmacies, independent physicians and NGOs.

iv. **Health care functions (HC)**: Types of goods and services provided and activities performed within the RH sub-accounts boundary, e.g., curative care, preventive, rehabilitative and long-term nursing care etc. Curative functions (out- and in = patient) and prevention and public health functions are described as follows:

i. Outpatient curative care: This includes outpatient curative maternal health services (antenatal and postnatal); family planning services (family planning counseling, method application and follow up, infertility treatment), reproductive health services (diagnosis and treatment of sexually transmitted infections, diagnosis and treatment of reproductive tract infections, diagnosis and treatment of reproductive health-related cancers), ambulatory treatment of gynaecological problems;

ii. Inpatient curative care: This function includes maternal health services (obstetric services for complicated deliveries and emergencies, ancillary services such as laboratory tests and pharmaceuticals); family planning services (sterilizations, ancillary services and pharmaceuticals, abortion services); reproductive health services (diagnosis and treatment of reproductive tract infections, diagnosis and treatment of reproductive health-related cancers, ancillary services and pharmaceuticals); and

iii. Prevention and public health services: Under this function are included maternal health preventive programmes, family planning programmes, prevention of reproductive health-related communicable and non-communicable diseases)

### Limitations

The study has the limitations below, which have to be taken into consideration when interpreting the results.

As a result of lack of disaggregated data from the respondents, ratios were used to split government in-facility spending on RH by using utilization data from the Health Facility Census.

The study did not undertake a survey on providers; therefore, information included on providers was generally based on assumptions and ratios, as described in the data analyses section.

Insurance scheme data on RH-specific expenditures were often insufficient for the purposes of NHA.

No recent costing studies of RH services exist.

Given the lack of data, it was not possible to distinguish between family planning and maternal health spending within the RH subaccount.

A household survey was not conducted; data used were from the 2003/04 Namibia Household Income and Expenditure Survey.

## Results

### RH financing indicators

RH expenditure declined in relative as well as absolute terms between the two financial years 2007/08 and 2008/09. RH expenditure per woman of reproductive age decreased from US$ 148 in 2007/08 to US$ 126 in 2008/09. General RH financing indicators are depicted in Table 2 below.

**Table 2:** RH financing indicators

Indicator	Financial year	
	
	2007/08	2008/09
RH expenditure per woman of reproductive age (US$)	148	126
RH expenditure as a percentage of GDP	0.8	0.7
RH expenditure as a percentage of total health expenditure	12.4	10.3
OOP spending on RH per woman of reproductive age (US$)	4.6	4.5
OOP spending as a percentage of TRHE	3.1	3.6

Although RH expenditure per woman of reproductive age declined substantially between the two financial years, the amount is still higher than what is observed in the majority of African countries.

### RH financing sources

**Figure 1:**
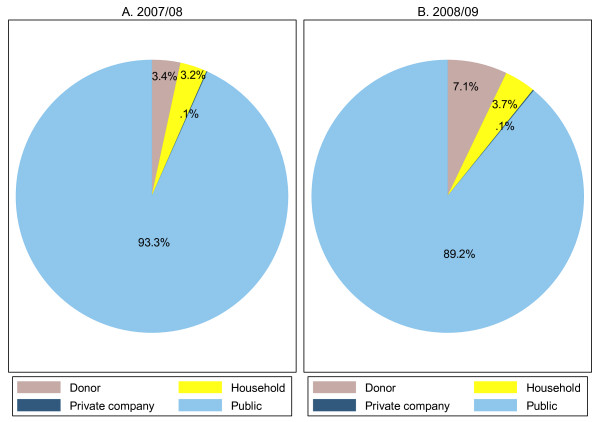
**RH financing sources, FY 2007/08-FY 2008/09**.

The lion's share of the RH expenditure was obtained from public sources. The share of public sources decreased by 4 percentage points in 2008/09 compared to the level in 2007/08. Figure 1 below presents the share of each source in RH financing over the two financial years.

It can be discerned from Figure 1 that donor funding for RH more than doubled in FY 2008/09 as compared to its share in 2007/08. Similarly contribution of households manifested an increase of about 15% in 2008/09 as compared to the 2007/08 levels.

### Household RH expenditure

**Figure 2:**
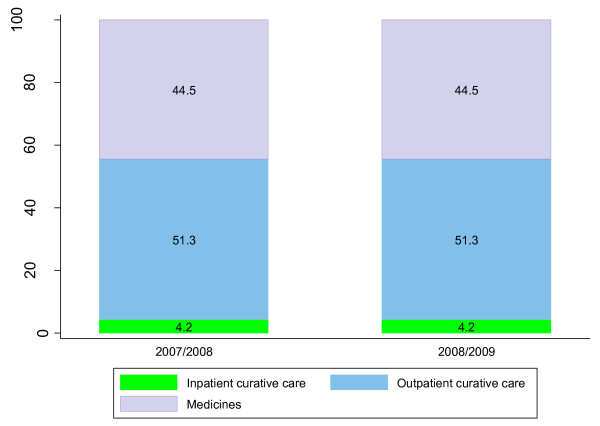
**Distribution of out-of-pocket RH spending by RH care functions**.

Out-of-pocket expenditure accounted for about 97% of household expenditure on reproductive health, implying that OOP expenditure constituted about 3.1% and 3.6% of the total reproductive health expenditure in 2007/08 and 2008/09 respectively. Although there is a slight increase in relative terms, the OOP expenditure was constant in absolute terms-about US$ 4.5 in both financial years. The OOP reproductive health expenditure was mainly paid to private clinics and dispensing chemists-both accounting for about 76% of total OOP in both financial years. About 3% of the out-of-pocket RH funds were spent in the offices of traditional healers. Furthermore, about 95% of OOP spending on RH was used to purchase outpatient curative care and prescription and over-the-counter medicines. Figure 2 below depicts the distribution of OOP RH expenditures by Reproductive health care functions.

### RH financing agents

**Figure 3:**
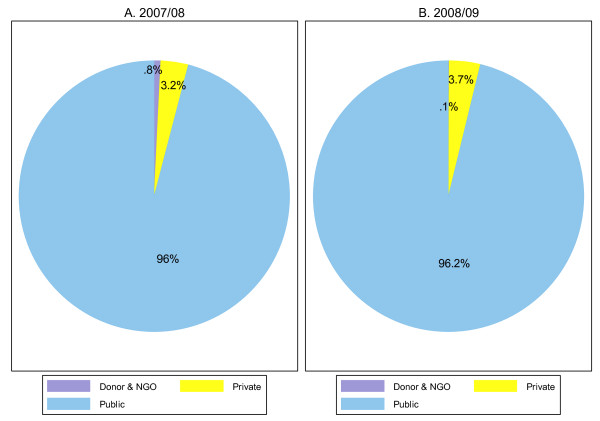
**RH financing agents, FY 2007/08-FY 2008/09**.

About 96% of the RH funds were managed by public entities during both financial years. These public entities included the Ministry of health and Social Services, Ministry of Gender Equality and Child Welfare, Ministry of Regional, Local Government and housing, Ministry of Defense and Public Service Employees Medical Aid Scheme (PSEMAS). However, it has to be noted that the MOHSS was the dominant financing agent managing about 90% of the RH funds. Figure 3 presents the distribution of financing agents for RH expenditures.

As managers of RH funds, donors and NGOs and household played a minor role-both entities accounting for only 4% of the TRHE.

### Distribution of RH expenditure by health care providers

**Figure 4:**
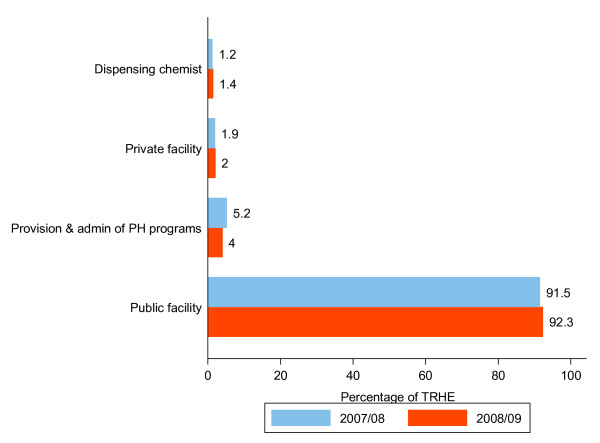
**Distribution of RH expenditure by health care providers, 2007/08-2008/09**.

RH spending at public facilities (public and mission hospitals, health centers, and clinics) accounted for more than 90 percent of TRHE for both FY 2007/08 and 2008/09, as shown in Figure 4.

Expenditures on private facilities and dispensing chemists together accounted for slightly more than 3 percent of TRHE in both financial years.

### Distribution of RH expenditure by function

**Figure 5:**
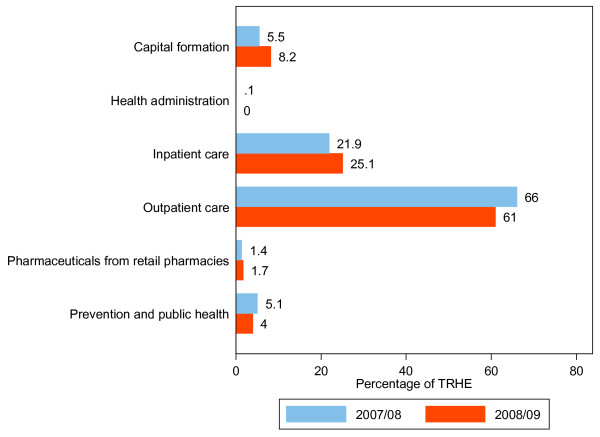
**Distribution of RH expenditure by function, 2007/08-2008/09**.

Curative care consumed the greatest proportion of RH expenditures in the two financial years. The share of prevention and public health services was not more than 5% during the period under review. Figure 5 depicts the distribution of RH expenditures over the two financial years.

As can be seen from Figure 5, outpatient curative care decreased by 5 percentage points, while a modest increase was observed in RH expenditure on inpatient care and capital formation.

### Discussion, conclusion and policy implications

This RH resource tracking exercise provides an overview of the flow of RH funds in the health sector. It provides information on the total volume of resources for reproductive health in Namibia and the flow among the different NHA entities-sources, financing agents, providers and health care functions.

Reproductive health spending of US$ 126 per woman of reproductive age compares very favorably as compared to those of countries such as Kenya (US$ 14) [14], Malawi (US$ 11.4) [15], Rwanda (US$ 8) [16]. The increasing trend in the maternal mortality ratio despite expenditure on reproductive health that is by far better than that of most countries in Africa may be an indication of inefficiency-both technical and allocative -, inequity in access to maternal health interventions and low quality of care. Earlier studies conducted have shown the presence of high degrees of technical inefficiency in hospitals that consume the bulk of the health sector resources and inequities in access to maternal health interventions [17,18]. To demonstrate the level of inequities, it is worthwhile to give some examples. The population average of the Caesarean section rate is over 12%, which is a good coverage. However, a regional breakdown indicates that three of the poorest regions have caesarean section rates of less than 5%, which is under-provision of the service. In contrast, in three other comparatively better off regions, the rate of Caesarean section is more than 15%, which implies over-provision of the service. The rate is also three times more in urban areas compared to rural settings (18). Thus, although the population averages depict a favourable coverage of the intervention, they mask the reality in that there are geographical localities where there is under-coverage. Hence, if deliberate actions are not undertaken to identify the causes for under-coverage and target resources to those whose unmet needs are high, greater expenditure by itself will not improve the increasing maternal mortality ratio.

The high degree of income inequality as measured by a Gini index of 74.3 is also an indication of the possible inequality in access to the social determinants of maternal health and access to maternal health interventions.

Similarly, a study on the technical efficiency of district hospitals has also shown high levels of technical inefficiency (17) implying high levels of wastage in resource use. Thus, in the presence of wastage, relatively high levels of expenditure on maternal health services may not bring about the desired change.

Out-of-pocket RH spending of 3.6% is far below the threshold of 15% beyond which the likelihood of catastrophic expenditure increases. Catastrophic expenditure is said to occur when households spend more than 40% of their disposable income after deducting subsistence allowances [19]. Thus, households are less likely to be impoverished as a result of out-of-pocket payments for reproductive health services. This should, however, be viewed cautiously, as out-of-pocket payment is not the only determinant of catastrophic expenditure. Other factors such as access and use of health services and ineffective mechanisms to pool financial risks are equally important [20] and need to be critically examined in the Namibian context.

In both financial years, government was the main source of RH funds (more than 89%). This is an indication of the high level of commitment of the Namibian Government to the health of women in their reproductive years. With due attention to the issues of efficiency, equity and quality mentioned above and addressing demand side problems that may deter utilization of services, the country would be able to reverse the trend of increasing maternal mortality ratio and accelerate its progress towards the achievement of the MDG 5 targets.

The Ministry of Health and Social Services is the main financing agent, managing more than 90% of the RH funds. The role of donors and NGOs as financing agents is minimal (below 1%). While donors were the source of about 7% of the RH funds in 2008/09, they only managed 0.1% of the RH funds as financing agents. This may perhaps indicate that most of donor finance for RH was pooled with the MOHSS' resources to avoid fragmentation and inefficiency.

Public providers are the main consumers of RH expenditure. RH expenditure on private providers was only 2%. Given the presence of a strong private sector in the country, it may be worthwhile to strengthen public-private partnerships in order to create synergy and maximize maternal health outcomes.

In conclusion, Namibia's expenditure on reproductive health is remarkable by the standards of Africa and other middle-income countries. However, an increasing maternal mortality ratio does not bode well with the level of reproductive health expenditure. It is therefore important to critically examine the state of efficiency in the allocation and use of reproductive health expenditures in order to improve health outcomes.

## Competing interests

The authors declare that they have no competing interests.

## Authors' contributions

TM, MS and NR led and conducted the NHA study and reviewed the article. JK and EZ did further analyses of the data and drafted the article. All authors read and endorsed the final manuscript.
